# Acute Viral Encephalitis Mimicking Idiopathic Intracranial Hypertension in an Immunocompetent Obese Adolescent Girl

**DOI:** 10.7759/cureus.51896

**Published:** 2024-01-08

**Authors:** Ramaning Loni, Abdulaziz H Khushaym, Abdulaziz B. Aldoseri, Ali Alfehaid, Gabriel Fox, Shatha Hasan

**Affiliations:** 1 Pediatrics, King Hamad University Hospital, Busaiteen, BHR

**Keywords:** increased intracranial pressure, varicella infection, headache, encephalitis, zoster

## Abstract

Varicella zoster virus (VZV) is common worldwide and one of the main causes of infectious encephalitis. We report the case of a 13‐year‐old girl who presented to the emergency department with complaints of recurrent episodes of vague headaches associated with photophobia multiple times within three days before hospitalization. The patient had a history of morbid obesity without other metabolic complications as well as a history of chicken pox in childhood. Doctors subsequently diagnosed her with increased intracranial pressure owing to suspected VZV encephalitis. A lumbar puncture confirmed the presence of VZV in the cerebrospinal fluid. After admitting the diagnosis of VZV encephalitis, doctors treated the patient with intravenous acyclovir and discharged her with oral acyclovir. This case highlights the importance of considering VZV encephalitis in the differential diagnosis for patients presenting with headaches, especially in the presence of typical vesicular skin rashes. The case has an unusual complication associated with shingles, which doctors diagnosed early and treated appropriately.

## Introduction

Varicella zoster virus (VZV) is one of the common herpes viruses known to cause infection in humans. VZV causes one of two infections: herpes simplex (chicken pox) and reinfection after treatment for chicken pox, which can cause herpes zoster (shingles). After primary VZV infection, patients pick up a latent VZV in the cranial nerve and dorsal root ganglia [1]. People with herpes zoster commonly present with a skin rash in one or two adjacent dermatomes. The skin rash usually appears on the trunk along a thoracic dermatome or on the face, and it usually does not cross the body’s midline. The skin rashes are painful, itchy, or tingly. A child can experience any of these symptoms for several days before the skin rash starts like headache, photophobia (sensitivity to bright light), or malaise. The skin rash develops into clusters of vesicles. New vesicles continue to form over three to five days, and the rash progressively dries and scabs over. The skin rash usually heals within two to four weeks, and permanent skin discoloration and scarring can occur later [1].

T cells play a key role in immunity control, and reactivation of the virus usually occurs with aging or because of immunosuppression. This can cause acute neuritis and a vesicular rash usually distributed to the dermatomes in the sensory ganglion, which are the typical symptoms of zoster [2]. Other complications include skin and soft tissue infection, neurological complications including encephalitis, Reye’s syndrome, pneumonia, hepatitis, and many more [3]. The average mortality rate of VZV infection is 12-15%, which may be higher in the case of immunocompromised patients. VZV is one of the most common causes of infectious neurological diseases such as encephalitis and infectious meningitis [4]. The examination of cerebrospinal fluid (CSF), neurological symptoms, imaging abnormalities, and clinical manifestations all contribute to the diagnosis. Doctors administer antivirals as part of the management, which has the potential to result in a full recovery [5].

VZV encephalitis is one of the most serious complications of VZV infections, although only one in 33,000-50,000 people with VZV develop encephalitis [6], accounting for around 20% of varicella-related hospitalization [7]. There are two distinct forms of encephalitis: acute cerebellar ataxia and diffuse encephalitis. These symptoms typically develop within the first week of infection but can be the presenting symptom. A case series done in August 2012 showed that 55% of patients had VZV encephalitis but had no cutaneous rash before hospitalization [8]. Acute cerebellar ataxia typically develops in children but usually has a limited time course and ends with full recovery, whereas diffuse encephalitis most often occurs in adults, with symptoms of delirium, focal neurological signs, and seizures. Encephalitis is one of the rare complications of VZV infection according to the World Health Organization. It has a relatively poor prognosis as compared to other extracutaneous complications of VZV [9].

## Case presentation

A 13-year-old girl with astigmatism and wearing glasses presented with a four-day history of worsening headaches. The patient, who was morbidly obese with a BMI of 36, presented at the emergency department (ED) three times in the preceding three days with the same complaint. The headache, originating in the frontal region and radiating to the whole head, was associated with photophobia and partial response to paracetamol. The headache mostly occurred in the early morning hours and worsened whenever the patient bent forward. The headache disturbed her sleep. She also reported nausea, dizziness, and four episodes of nonbilious, nonprojectile vomiting following meals that started one day before the last ED visit. There was no associated history of fever, cold, cough, or neck stiffness; no hearing or visual disturbances (no diplopia, no nystagmus) except sensitivity to bright light; and no other neurological deficits such as weakness in any part of the body or any abnormal body movements. There was no history of urinary or bowel changes.

At her first presentation, the patient had a follow-up ophthalmology appointment for her astigmatism and possible other refractive irregularities. When personnel in the pediatric ophthalmology outpatient department (OPD) took her vitals, they found that the patient had bradycardia (35-40 beats per minute) and immediately referred her to the ED. Doctors administered symptomatic treatment, including oral diclofenac, paracetamol, and omeprazole as stat medicines. Following the patient’s ophthalmology appointment, medical personnel discharged her pain-free. On the second visit (second day), she repeated the same complaints she made to the ED and underwent some blood tests and a CT brain scan, which reported no significant acute findings except for a small right maxillary retention cyst. Medical personnel again discharged the patient. However, during her third visit for a pediatric OPD follow-up on October 11, 2023, the pediatric team noticed the patient’s bradycardia and hypotension, leading to its decision to send her immediately to the ED.

Upon the ED pediatric team’s first evaluation and primary and secondary assessments, the patient displayed signs of discomfort, photophobia, and drowsiness, with a blood pressure of 80/50 mmHg and a heart rate of 40 beats per minute. The ECG showed sinus bradycardia. The team started high-flow oxygen therapy and administered a bolus of normal saline to correct the hypotension and raise the blood pressure to 106/60 mmHg. A pediatric intensivist’s further assessment and review along with a CT brain scan revealed signs of cerebral edema owing to the presence of small ventricles, effacement of sulci and gyri, and loss of gray and white matter differentiation. The team made a provisional diagnosis of increased intracranial pressure (ICP) owing to idiopathic intracranial hypertension associated with obesity or an underlying CNS infection. The team gave the patient a bolus of 3% saline and transferred her to the pediatric ICU for further management.

The patient’s medical history was unremarkable except for her history of chicken pox during infancy. She did not receive the varicella vaccine during childhood but was up to date with all other vaccines.

**Figure 1:**
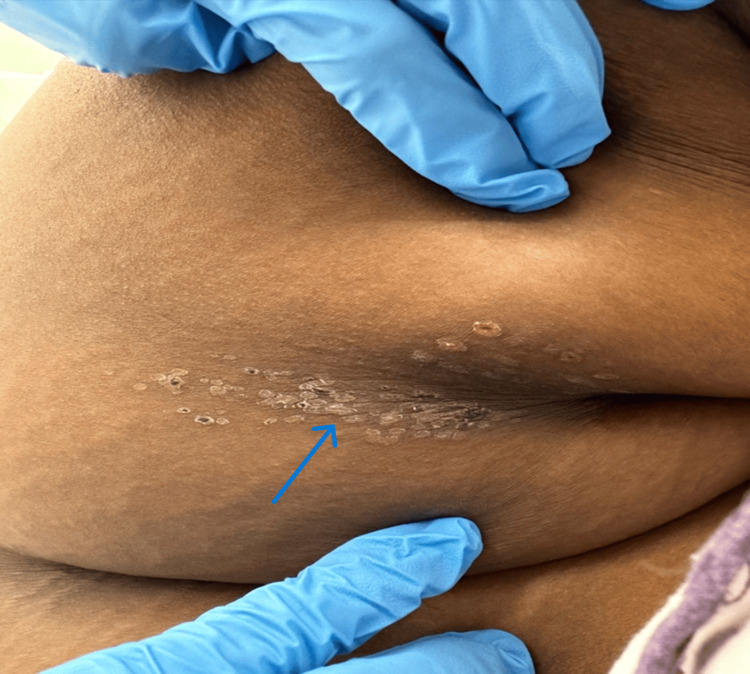
Vesicular lesions (crusted) on the patient’s back

**Figure 2:**
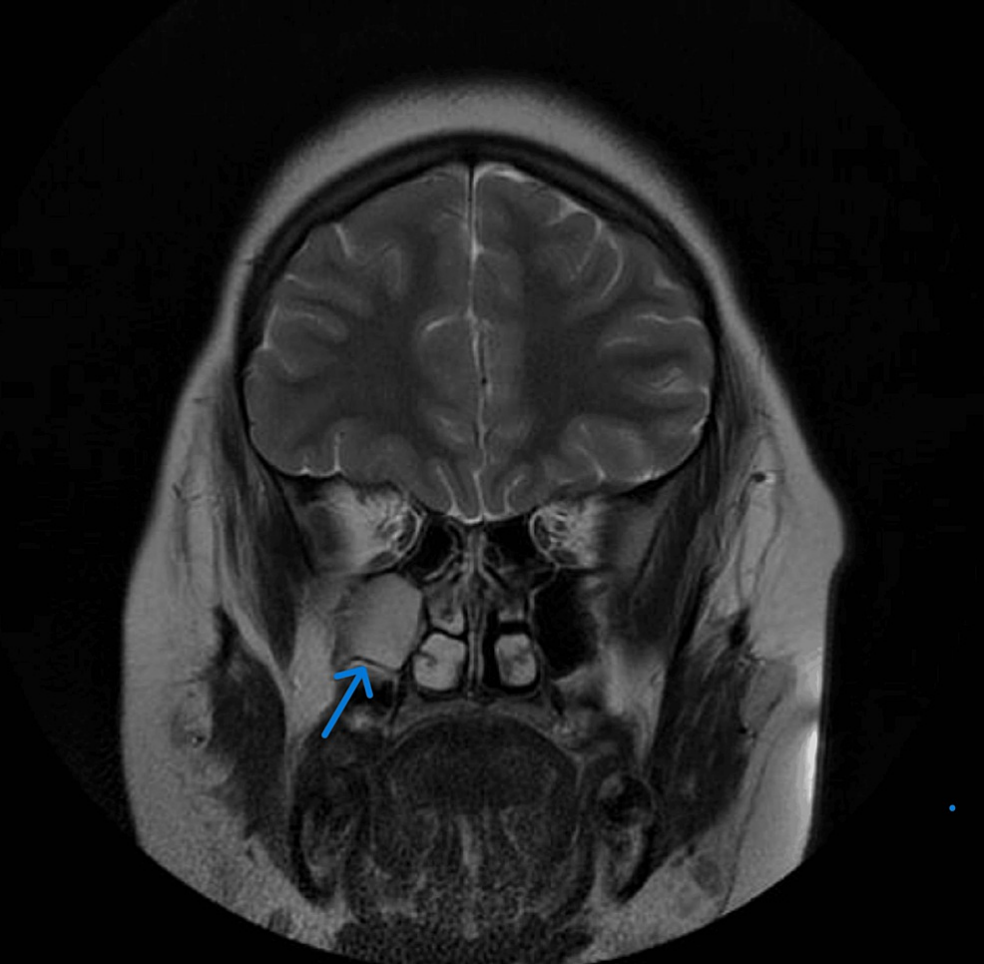
MRI brain scan with T2-weighted image, a sagittal section with a 3D view showing the right maxillary retention cyst

The patient received intravenous fluids and hyperosmolar therapy such as 3% saline, and she received injected ceftriaxone upon admission. In the pediatric ICU, a pediatric neurologist examined the patient and ordered a lumbar puncture and MRI brain scan with magnetic resonance venography. A pediatric anesthetist performed the lumbar puncture on the second day of pediatric ICU admission under hyperosmolar therapy. During the lumbar puncture procedure, the team observed an elevated opening pressure (> 30 cm H2O). CSF routine analysis revealed a total white blood cell count of 1,300 cells (100% lymphocytes with 100 red blood cells) and normal glucose and protein levels (Table 1), which goes in favor of viral meningoencephalitis or viral encephalitis. The meningitis or encephalitis panel was positive for VZV (Table 2). The team confirmed a diagnosis of VZV CNS infection (VZV encephalitis) (Table 2) and started therapeutic doses of IV acyclovir. The MRI and MRA were unremarkable, except for a right maxillary retention cyst (Figure 1) and left azygous vein hypoplasia (Figure 2), ruling out meningeal enhancement or signs of ICP. When the team asked the patient about any vesicular lesions on her abdomen or back, she replied that a lesion had recently appeared on her back. She had attended an infectious disease consultation, and the consulting doctor advised her to continue antivirals for 14 days for VZV viral encephalitis and thereafter to undergo tests for immunodeficiency. The patient continued to receive ceftriaxone for seven days until all cultures were available as well as 3% saline osmotic therapy for ICP management for five days (tapering doses). An immunology workup to rule out immunodeficiency yielded normal results. The patient was discharged on October 19, 2023 (after one week of hospitalization), and she was continued with oral acyclovir for an additional seven days.

**Table 1:** CSF laboratory findings

Parameter	Findings
Glucose (CSF)	3.18 mmol/L (2.2:3.9)
Protein Total (CSF)	82.28 mg/dL (15:45)
Red Blood Cells (CSF)	150 cells/c.mm
White Blood Cells (CSF)	1300 cells/c.mm
CSF Culture	Negative
CSF Gram Stain	No growth detected

**Table 2:** Film array meningitis or encephalitis panel

Film Array Meningitis/Encephalitis Panel	
E. coli K1	Not Detected
Streptococcus agalactiae	Not Detected
Streptococcus pneumoniae	Not Detected
Enterovirus (EV)	Not Detected
Herpes simplex virus 1	Not Detected
Herpes simplex virus 2	Not Detected
Human herpesvirus 6	Not Detected
Cryptococcus neoformans/gattii	Not Detected
Human par echovirus	Not Detected
Varicella zoster virus (VZV)	Detected
Cryptococcus neoformans/gattii	Not Detected
Hemophilus influenza	Not Detected
Listeria monocytogenes	Not Detected
Neisseria meningitides	Not Detected
Cytomegalovirus (CMV)	Not Detected

Film Array Meningitis/Encephalitis Panel	
E. coli K1	Not Detected
Streptococcus agalactiae	Not Detected
Streptococcus pneumoniae	Not Detected
Enterovirus (EV)	Not Detected
Herpes simplex virus 1	Not Detected
Herpes simplex virus 2	Not Detected
Human herpesvirus 6	Not Detected
Cryptococcus neoformans/gattii	Not Detected
Human par echovirus	Not Detected
Varicella zoster virus (VZV)	Detected
Cryptococcus neoformans/gattii	Not Detected
Hemophilus influenza	Not Detected
Listeria monocytogenes	Not Detected
Neisseria meningitides	Not Detected
Cytomegalovirus (CMV)	Not Detected

Film Array Meningitis/Encephalitis Panel	
E. coli K1	Not Detected
Streptococcus agalactiae	Not Detected
Streptococcus pneumoniae	Not Detected
Enterovirus (EV)	Not Detected
Herpes simplex virus 1	Not Detected
Herpes simplex virus 2	Not Detected
Human herpesvirus 6	Not Detected
Cryptococcus neoformans/gattii	Not Detected
Human par echovirus	Not Detected
Varicella zoster virus (VZV)	Detected
Cryptococcus neoformans/gattii	Not Detected
Hemophilus influenza	Not Detected
Listeria monocytogenes	Not Detected
Neisseria meningitides	Not Detected
Cytomegalovirus (CMV)	Not Detected

Film Array Meningitis/Encephalitis Panel	
E. coli K1	Not Detected
Streptococcus agalactiae	Not Detected
Streptococcus pneumoniae	Not Detected
Enterovirus (EV)	Not Detected
Herpes simplex virus 1	Not Detected
Herpes simplex virus 2	Not Detected
Human herpesvirus 6	Not Detected
Cryptococcus neoformans/gattii	Not Detected
Human par echovirus	Not Detected
Varicella zoster virus (VZV)	Detected
Cryptococcus neoformans/gattii	Not Detected
Hemophilus influenza	Not Detected
Listeria monocytogenes	Not Detected
Neisseria meningitides	Not Detected
Cytomegalovirus (CMV)	Not Detected

Film Array Meningitis/Encephalitis Panel	
E. coli K1	Not Detected
Streptococcus agalactiae	Not Detected
Streptococcus pneumoniae	Not Detected
Enterovirus (EV)	Not Detected
Herpes simplex virus 1	Not Detected
Herpes simplex virus 2	Not Detected
Human herpesvirus 6	Not Detected
Cryptococcus neoformans/gattii	Not Detected
Human par echovirus	Not Detected
Varicella zoster virus (VZV)	Detected
Cryptococcus neoformans/gattii	Not Detected
Hemophilus influenza	Not Detected
Listeria monocytogenes	Not Detected
Neisseria meningitides	Not Detected
Cytomegalovirus (CMV)	Not Detected

During the patient’s follow-up appointment two weeks later, the pediatric ICU consultant evaluated her condition. Overall, it was good, with a Glasgow coma scale of 15/15, a temperature of 36.5°C (tympanic), a heart rate of 87 beats per minute, a weight of 99.3 kilograms, a height of 154.2 centimeters, an oxygen saturation level of 99.0%, and BMI of 41.76 kg/m². Her blood pressure was recorded as 120/60 mmHg, and a neurological examination revealed normal functioning. She did not have headaches or changes in vision or hearing, and her gait was normal. The mother reported that the patient resumed attending school without any issues.

The pediatric ICU consultant informed the patient about the red flags like severe headache, vomiting, unsteadiness of the gait, or weakness in any part of the body. The consultant advised her to seek immediate medical attention at the ED and recommended that she visit a dietician and obesity clinic for weight reduction and lifestyle modifications.

## Discussion

VZV encephalitis can sometimes be a challenging condition to diagnose and often leads to delayed treatment and adverse patient outcomes. Pediatricians should be well versed in clinical features and risk factors with high suspicion and low threshold for prompt diagnosis of VZV encephalitis. With increased awareness, pediatricians’ early detection and faster treatment can decrease the risk of complications and improve the patient’s quality of life, as our good approach in this case demonstrated. Therefore, international guidelines recommend initially following the approach whenever doctors suspect a serious illness or injury, regardless of the underlying cause [9,10].

**Figure 3:**
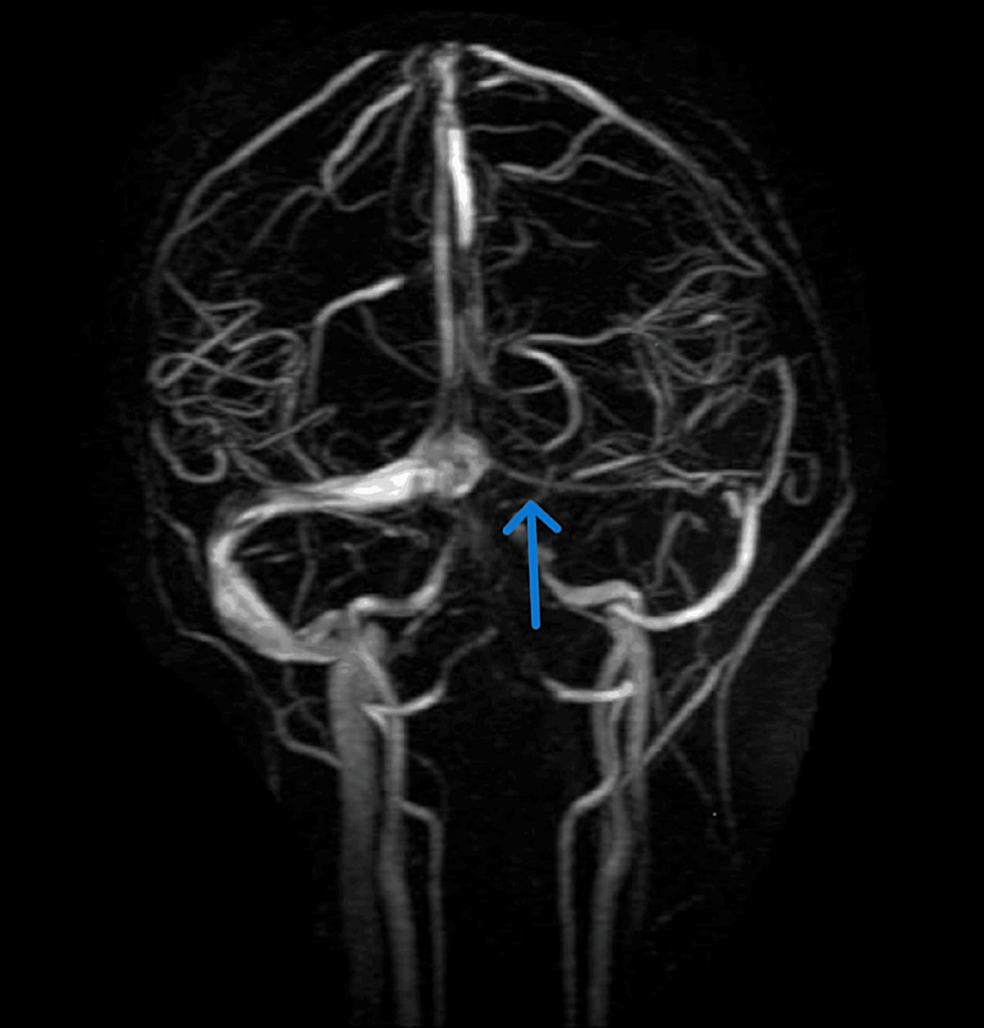
MRA brain scan showing hypoplasia of the left transverse sinus, otherwise normal findings

Researchers recommend the airway, breathing, circulation, disability, exposure (ABCDE) approach, a widely accepted, expert-based algorithm for the management of potentially critically ill or injured patients of all ages [10,11]. The exposure element is essential, particularly where the presence of typical vesicular rashes can help diagnose cases of suspected VZV encephalitis and where failure to see the cysts could result in misdiagnosis (Figure 3). The approach works like a stepwise algorithm, which enables all healthcare providers, including pediatricians, to find any critical conditions and respond to them based on priority [11,12]. The research shows that there is a meaningful relationship between adherence to the ABCDE approach and improved assessment and initial treatment of patients in need of emergency care [10,11]. Severe disseminated VZV can be present as hepatitis, encephalitis, pneumonitis, and generalized rash over the body. However, the delay or absence of the dermatological features associated with VZV is usually rare (Figure 3), and there are many reported cases of encephalitis developed before the presentation of a rash or even no rash [11,12]. Our patient’s underlying obesity with acute headache, nausea, and vomiting (which occurred mostly in the morning) along with photophobia showed signs of ICP, thus indicating idiopathic intracranial hypertension, especially when she was afebrile and did not reveal skin lesions. The imaging findings in our study were either normal or showed nonspecific abnormalities such as cortical atrophy in a few cases, and there was no evidence of stroke. However, compared to our case’s MRI, it was unremarkable except for a right maxillary sinus retention polyp (Figure 1) and hypoplasia of the left transverse sinus as incidental findings (Figure 2).

Regarding the clinical presentation of index cases as compared with multiple reported cases, De Broucker et al. [8] investigated a series of cases of acute VZV encephalitis in 20 patients. The researchers found that the two patients (one child and one adult) had neurological symptoms associated with VZV encephalitis, despite no longer having cutaneous signs of the infection upon admission. This condition, known as zoster sine herpete, occurs when neurological symptoms of VZV infection manifest without a visible rash. In these cases, detecting VZV DNA or specific VZV antibodies in the CSF is the only proof of VZV involvement (Table 2).

Furthermore, of the 20 patients studied, the researchers identified VZV in 16 patients using PCR-based viral DNA testing in the CSF. The remaining four cases occurred during or just after the VZV rash developed. Nine cases had a rash, and 18 cases developed acute encephalitis syndrome, which included fever and other symptoms such as diffuse brain dysfunction, focal neurological symptoms, seizures, and cranial nerve palsy. This suggests that most patients in the study had encephalitis as a clinical presentation as seen in our case.

In De Broucker et al.’s study [8], all patients received acyclovir in different doses and at various times, but the case fatality rate remained high (about 15% in all patients), and some neurological sequelae appeared in children at discharge or during their three-year follow-up.

## Conclusions

A detailed history and examination of the patient are important to detect red flags for making an early diagnosis and devising proper management. The red flags in headache history include acute onset, severe headache, early morning headache, and headache that disturbs sleep. It is therefore important to look for the organic cause of the headache. VZV encephalitis can occur in immunocompetent children without typical onset with fever. It is, therefore, important to maintain a high index of suspicion.
